# Investigation of Formation Behaviour of Al–Cu Intermetallic Compounds in Al–50vol%Cu Composites Prepared by Spark Plasma Sintering under High Pressure

**DOI:** 10.3390/ma14020266

**Published:** 2021-01-07

**Authors:** Dasom Kim, Kyungju Kim, Hansang Kwon

**Affiliations:** 1 Department of Materials System Engineering, Pukyong National University, Busan 48547, Korea; dasom.kim@f.mbox.nagoya-u.ac.jp; 2 The Industrial Science Technology Research Center, Pukyong National University, Busan 48547, Korea; ngm13@ngm.re.kr; 3 Department of R & D, Next Generation Materials Co., Ltd., Busan 48547, Korea

**Keywords:** aluminium (Al), copper (Cu), metal matrix composites, ball milling, spark plasma sintering (SPS), intermetallic compounds (ICs), thermal conductivity

## Abstract

Al–Cu matrix composites with excellent mechanical and thermal properties are among the most promising materials for realising high performance in thermal management systems. However, intermetallic compounds (ICs) formed at the Al/Cu interfaces prevent direct contact between the metals and severely deteriorate the thermal conductivity of the composite. In this study, we systemically investigated the formation behaviour of Al–Cu ICs as a function of compaction pressure at a low temperature of 380 °C. The phases of the Al–Cu ICs formed during sintering were detected via X-ray diffraction, and the layer thickness and average area fraction of each IC at different compaction pressures were analysed via micro-scale observations of the cross-sections of the Al–Cu composites. The ICs were partially formed along the Al/Cu interfaces at high pressures, and the formation region was related to the direction of applied pressure. The Vickers hardness of the Al–Cu composites with ICs was nearly double those calculated using the rule of mixtures. On the other hand, the thermal conductivity of the composites increased with compaction pressure and reached 201 W·m^−1^·K^−1^. This study suggests the possibility of employing Al–Cu matrix composites with controlled IC formation in thermal management applications.

## 1. Introduction

High heat dissipation and low weight are the requisite properties of components used in electric devices, such as semiconductors, displays, batteries, and motors, owing to the trends of transportation electrification (electric and hybrid automobiles, marine vehicles, and airplanes) and device miniaturisation. Metal matrix composites with high yield and tensile strength and thermal conductivity are considered excellent thermal management materials [1,2,3]. Among metals, Cu [4,5,6] and Al [7,8,9] have been primarily used as metal matrices owing to their high thermal conductivities.

Al–Cu matrix composites are among the most suitable materials for use in thermal management owing to their high specific strength and thermal conductivity and have recently attracted considerable attention. Studies on Al–Cu matrix composites reinforced with SiC [10] or diamond [11,12], carbon nanotubes (CNTs) [13,14,15], and graphene [16] and displaying superior thermal properties have been reported in recent decades. Uniform dispersion of the nano-sized fibres in the matrix is important for the high performance of the composite materials. This is achieved using powder metallurgical processes, which can yield metal matrices with high surface area fractions. However, the thermal conductivity deteriorates significantly owing to the high fraction of the Al/Cu interface being occupied by the Al–Cu intermetallic compounds (ICs) formed. The thermal conductivity decreases with an increase in the Cu content of the Al matrix [11], which limits the suitable composition range of Al–Cu matrix composites. Although ICs have poor electrical and thermal conductivities, they can facilitate strong bonding between Al and Cu via chemical reaction. Therefore, the control of ICs to realise high thermal conductivity and strong interfacial bonding simultaneously is considered one of the most important factors in the fabrication of Al–Cu matrix composites.

In addition to the formation degree of ICs, the significant difference between the densities of Al (2.7 g·cm^−3^) and Cu (8.9 g·cm^−3^) is problematic in the preparation of Al–Cu composites with high Cu contents. In our previous study, Al–Cu composites with 20, 50, and 80 vol% Cu were fabricated via ball milling and spark plasma sintering (SPS). First, the optimum ball-milling conditions were determined and an encapsulated Al–Cu composite powder (with Cu particles surrounded by Al particles) was successfully prepared [17], then the Al-Cu composite was prepared by the SPS process. Sintering using direct or alternating current directly passing through the powder materials inside of a mould has been called SPS, the field assisted sintering technique (FAST), and pulse electric current sintering (PECS). Since its particles are sintered by joule heating at the contact point, the sintering can be conducted quickly compared to general sintering. Then, interfaces of different materials can be controlled. During SPS at 510 °C under 50 MPa, ICs were formed at the Al/Cu interface and completely surrounded the Cu particles. Hence, the Al–50vol%Cu composite, which had the highest Al/Cu interface area fraction, exhibited the least thermal conductivity, which was half that of Al. To improve the thermal conductivity of the Al–Cu composites, it is necessary to control the formation of ICs during sintering.

Generally, ICs are formed via thermal diffusion when heat is applied. A recent study revealed that ICs were partially formed at the interface between Al and Cu foils owing to the non-uniform accumulation degree of dislocations achieved via cold rolling (uniaxial high pressing) [18,19]. As the partial formation of ICs can enable direct contact between the metals at the interface, this approach was considered suitable for preventing the deterioration in thermal conductivity with an increase in the Al/Cu interface area. However, Al and Cu might be bonded weakly in the absence of heat or excessive stress could accumulate, which could additionally deteriorate the thermal conductivity. Moreover, the formation behaviour of ICs under the simultaneous application of heat and pressure has not yet been investigated.

In this study, we aimed to investigate the formation behaviour or mechanism of ICs under heat and pressure to realise high thermal conductivity. The SPS process, which involves the direct sintering of particles by plasma, was selected to conduct uniaxial compaction under high pressure with the simultaneous application of heat. The Al–50vol%Cu composites were fabricated via ball milling followed by low-temperature high-pressure SPS. The effects of compaction pressure on the formation behaviour of ICs at the Al/Cu interface during SPS were investigated by observing the microstructures and measuring the thickness and area fraction of each IC layer. The Vickers hardness values were measured to evaluate the effects of the ICs on the mechanical properties of the composites. Finally, the thermal conductivity of the composites was evaluated to determine the effect of the formation behaviour of ICs on the mechanism of thermal conduction.

## 2. Materials and Methods

### 2.1. Preparation of Al–Cu Composite Powder via Ball Milling

Pure spherical Al and Cu powders (99.9%, Metalplayer Co., Ltd., Incheon, Korea) with an average particle size of approximately 45 μm each were used as the raw materials. They were poured into a stainless-steel container with a ZrO_2_ ball (15 mm in diameter) and a stainless-steel ball (3.2 mm in diameter). The powder-to-ball volume ratio was 1:3. After the powders and the ball were loaded, 20 mL of heptane was added as a process control agent to prevent reaction with air during mechanical ball milling (SMBL-6, SciLab Mix^TM^, Programmable Ball Mill, Seoul, Korea) at a speed of 300 rpm for 12 h. The heptane was evaporated naturally in a fume hood after the milling was completed. The raw powders and their mixture as well as the ball-milled composite powder were observed via scanning electron microscopy (SEM, VEGA II LSU, TESCAN, Brno, Czech Republic), field emission scanning electron microscopy (FE-SEM, MIRA 3 LMH In-Beam, TESCAN, Czech Republic), and energy dispersive X-ray spectroscopy (EDS, EX-400, HORIBA, Kyoto, Japan). A cross-section of the composite powder, prepared via hot mounting and polishing, was observed via field emission electron probe micro-analysis (FE-EPMA, JXA-8530F, JEOL Ltd., Tokyo, Japan) to analyse its mixing state. The phases were detected via X-ray diffraction (XRD, Ultima IV, Rigaku, Tokyo, Japan) with a Cu Kα radiation source (λ = 1.5148 Å, 40 kV, and 40 mA) in the 2θ scanning range of 20–80°.

### 2.2. Fabrication of Al–Cu Composites via SPS

The composite powder was poured into a WC-Co mould with a square shape (10 mm × 10 mm), which was installed in the SPS equipment (SPS-321Lx, Fuji Electronic Industrial Co., Ltd., Tsurugashima, Japan). The temperature was monitored by thermocouple inserted into the hole of the mould. The samples were sintered at 380 °C at a heating rate of 30 °C·min^−1^ under 50, 100, 200, and 250 MPa for 5 min. The atmosphere during sintering was controlled to a vacuum state under 0.8 Pa.

### 2.3. Characterisation of Al–Cu Composites

The microstructures of the sintered Al–Cu composites were analysed using XRD, FE-SEM, and EDS. Additionally, EDS line scans were conducted along the Al/Cu interfaces to analyse the ICs. Digital image analysis was performed using ImageJ 1.52a software (NIH Image, US National Institutes of Health, http://rsb.info.nih.bov) to measure the area fraction of each phase in the Al–Cu composite. The density of the composite material was measured using the Archimedes method. The theoretical density was calculated using the rule of mixtures. The Vickers hardness (HM-101, Mitutoyo Corp., Kawasaki, Japan) was measured using a load of 0.3 kg for 15 s according to the JIS B 7725 and ISO 6507-2 standards. To evaluate the thermal conductivity, heat diffusivity and heat capacity were measured at room temperature (20 ± 2 °C) using a laser flash device (LFA467, Netzsch, Selb, Germany) according to the ISO 22007-4, ISO 18755, and ASTM E 1461 standards. The accuracy of the measuring device was ±3% for the heat diffusion coefficient and ± 5% for heat capacity.

## 3. Results and Discussion

**Figure 1:**
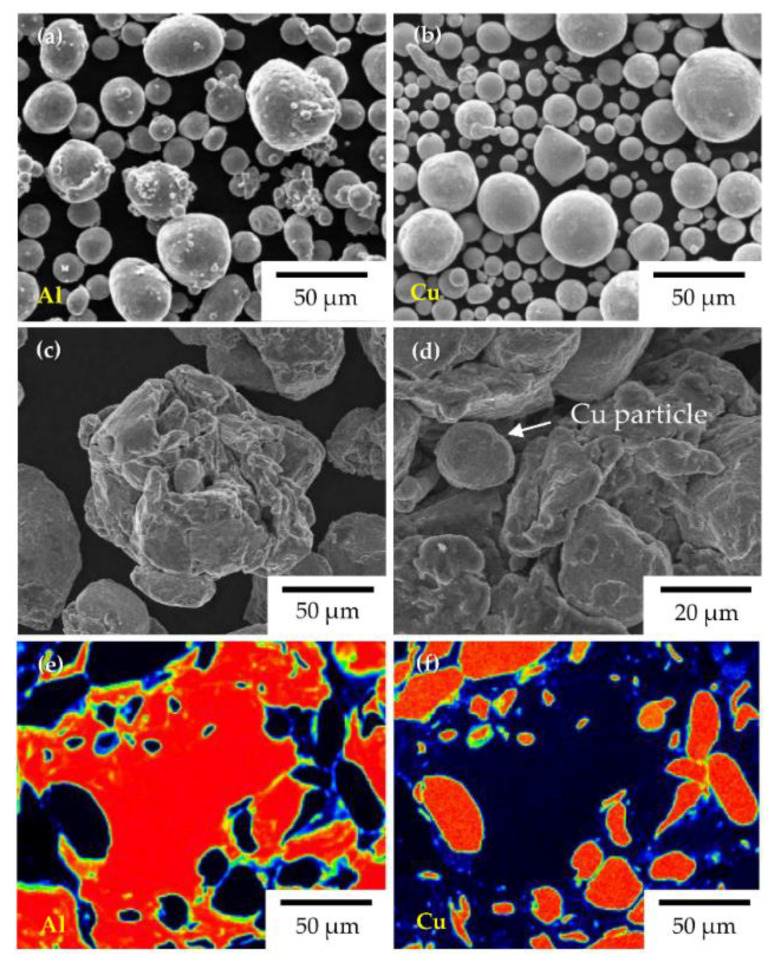
SEM micrographs of (**a**) raw Al powder, (**b**) raw Cu powder, and (**c**) Al–50vol%Cu composite powder prepared via ball milling; (**d**) high-magnification of (**c**); and EPMA mapping results of (**e**) Al and (**f**) Cu in a single particle of Al–50vol%Cu composite powder.

Figure 1 shows the morphology of the raw powders of Al and Cu and their ball-milled mixture. The raw powders of Al (Figure 1a) and Cu (Figure 1b) had clean surfaces and spherical particles with a particle size of approximately 40–50 μm, which corresponded to a mean particle size of 45 μm. As seen in Figure 1c, the Al–50vol%Cu composite powder had particles with rough surfaces and the particle size exceeded 50 μm as a result of coarsening during ball milling. The particles were coarsened because the Al particles with high ductility adhered owing to mechanical energy. The smaller, spherical particles with rather angled surfaces were believed to be Cu particles, which were crushed, and not deformed or caused to adhere, under the action of mechanical energy, as shown in Figure 1d. The coarsened particles resulting from ball milling were observed using FE-EPMA, which revealed them to be a combination of Cu and Al. The refined Cu particles, which were likely generated via crushing, were surrounded by Al particles without any pores, as seen from the EPMA mapping results of elemental Al (Figure 1e) and Cu (Figure 1f). The mixture of Al and Cu powders formed the Al–Cu composite powder, wherein each particle comprised both Al and Cu despite the significant difference between their masses. This enhanced the uniformity of the powder in the bulk state.

**Figure 2:**
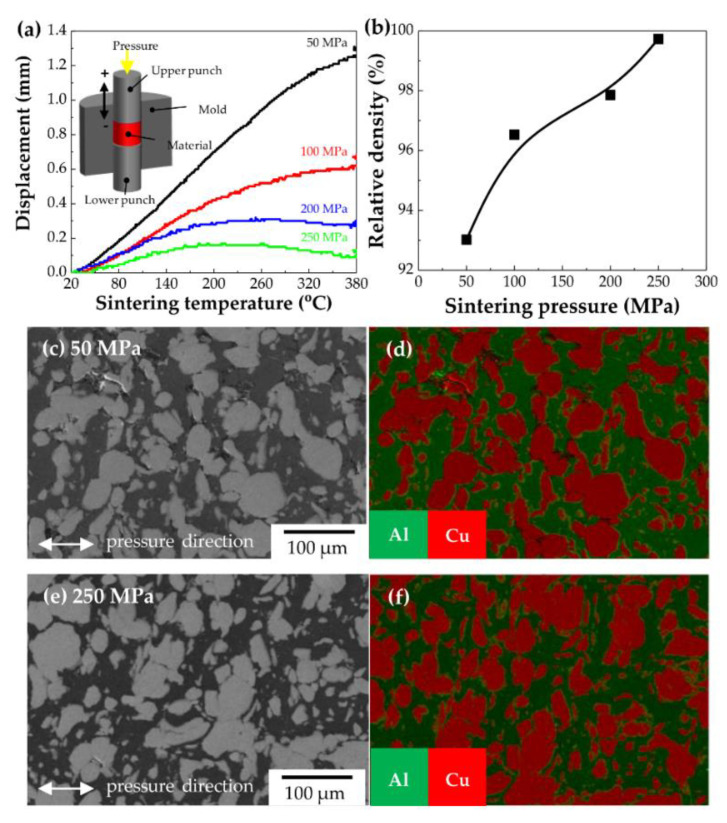
(**a**) Displacement profile as a function of temperature during the sintering of Al–50vol%Cu under various pressures; (**b**) relative density of Al–50vol%Cu composites as a function of compaction pressure, (**c**) FE–SEM micrograph, and (**d**) EDS mapping result of Al–50vol%Cu composite sintered under 50 MPa; and (**e**) FE–SEM micrograph and (**f**) EDS mapping result of Al–50vol%Cu composite sintered under 250 MPa.

The Al–Cu composite powder was sintered at 380 °C under pressure values of 50, 100, 200, and 250 MPa. Figure 2a shows the displacement profile as a function of the temperature recorded during sintering. The displacement increased with temperature, indicating that the powder expanded when heated, i.e., thermal expansion occurred during sintering. The degree of increase in displacement owing to heat decreased with the increase in pressure, indicating that the thermal expansion was suppressed by the compaction pressure. In addition, the displacement was saturated or even decreased (shrinkage) at higher compaction pressures and high temperatures. When materials are sintered, the stress from thermal expansion and compression as well as residual stress might be stacked within them. Therefore, greater amounts of stress might have accumulated at a compaction pressure of 250 MPa.

The relative density was calculated using the rule of mixtures; the theoretical densities of Al and Cu were 2.7 g·cm^−3^ and 8.9 g·cm^−3^, respectively. The relative density increased with compaction pressure and reached 100% at 250 MPa, as shown in Figure 2b. Densification decelerated in the pressure range of 100–200 MPa, but recovered thereafter in the pressure range of 200–250 MPa. This might have resulted from the different compaction behaviours of Al and Cu. To analyse the densification owing to pressure, cross-sections perpendicular to the direction of compression pressure were observed via FE-SEM and EDS of the Al–Cu composites sintered at 50 MPa (Figure 2c) and 250 MPa (Figure 2e). The bright and dark regions might be Cu and Al, respectively. The Cu particles were dispersed within the Al matrix, which was prepared by the deformation of the Al particles. This coincided with the EDS mapping results (Figure 2d,f). Pores partially residing at neat Al/Cu interfaces were observed in the composite sintered at 50 MPa but disappeared in the specimen sintered at 250 MPa, indicating an increase in the contact area between Al and Cu with pressure. This indicated that greater amounts of the Al–Cu ICs might have been formed during sintering at 250 MPa. To determine whether new phases were formed as well as their formation degrees, XRD analysis was conducted.

**Figure 3:**
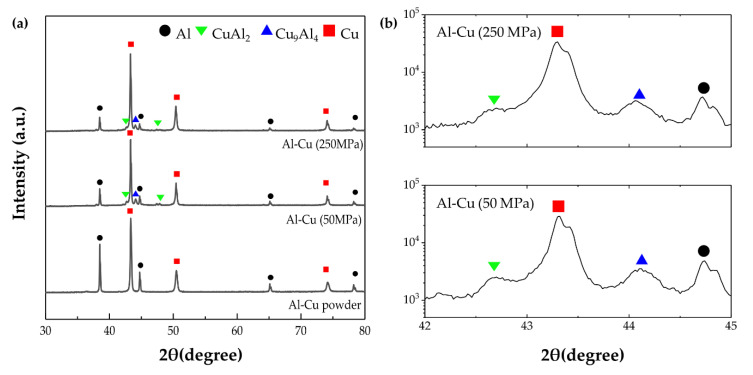
(**a**) XRD results of Al–50vol%Cu powder prepared via ball milling and of Al–50vol%Cu composites sintered at 380 °C under pressure values of 50 and 250 MPa, and (**b**) XRD results in the 2θ scanning range 42–45° of Al–50vol%Cu composites sintered at 50 and 250 MPa.

Figure 3 shows the XRD analysis results of the Al–Cu composite powder prepared via ball milling and the Al–Cu composites sintered at 380 °C under pressures of 50 and 250 MPa. The results showed that Al–Cu IC phases such as CuAl_2_ and Cu_9_Al_4_ were formed during sintering. Additionally, the intensity of the Al peak decreased drastically whereas that of the Cu peak remained rather constant (Figure 3a) after sintering, indicating that the Al was more consumed to form ICs than Cu. The peak intensity of Al, Cu, CuAl_2_, and Cu_9_Al_4_ in the range of 42–45° of the Al–Cu composite under 50 MPa and 250 MPa were compared in Figure 3b. With an increase in compaction pressure, the intensity ratio of Al/Cu was increased (0.11 to 0.17), but CuAl_2_ and Cu_9_Al_4_ were decreased (0.99 to 0.72). It indicates that the formation fraction of CuAl_2_ (Al-rich phase) was reduced under higher compaction pressure.

First, we considered the formation mechanism of the Al–Cu ICs. Although CuAl_2_, CuAl, Cu_4_Al_3_, and Cu_9_Al_4_ are present in the Al–Cu phase diagram, only CuAl_2_ and Cu_9_Al_4_ were detected in the present study. Similar results have been reported from previous studies on Al/Cu interfaces formed during hot rolling [20,21], extrusion [22], and welding [23,24], as well as in our previous study [17]. This can be explained on the basis of activation energy for growth. As the activation energies for the growth of CuAl_2_ and Cu_9_Al_4_ were much lower than those of the other phases under heat application [25], only these two phases were detected finally. When Al–Cu ICs are formed owing to heat energy, the thermal diffusion rate of Al in Cu is greater than that of Cu in Al [26]. Al is significantly consumed during the formation of the Al–Cu ICs, resulting in deep diffusion in Cu. Therefore, the layer of CuAl_2_ is thicker than that of Cu_9_Al_4_ when the ICs are formed by thermal diffusion. In Xu’s study, the activation energy for the growth of CuAl_2_ and Cu_9_Al_4_ was estimated. The activation energy (Q) was estimated in the Arrhenius equation substituting growth rate (D). The growth rate (D) was calculated with the empirical powder law, measuring each IC thickness depending on the annealing condition on a TEM micrograph. Since the activation energy for the growth of CuAl_2_ (60.66 kJ·mol^−1^) was lower than that of Cu_9_Al_4_ (75.61 kJ·mol^−1^), the CuAl_2_ layer was thicker than the Cu_9_Al_4_ layer.

When the compaction pressure was increased from 50 MPa to 250 MPa, the IC peak intensity of the Al–Cu composites decreased even though the area fraction of the Al/Cu interface increased owing to the removal of pores. This indicated that the ICs could be formed via different mechanisms under high pressure. To investigate the variation in IC formation with pressure, the interface with ICs was observed via FE-SEM with EDS line scan.

**Figure 4:**
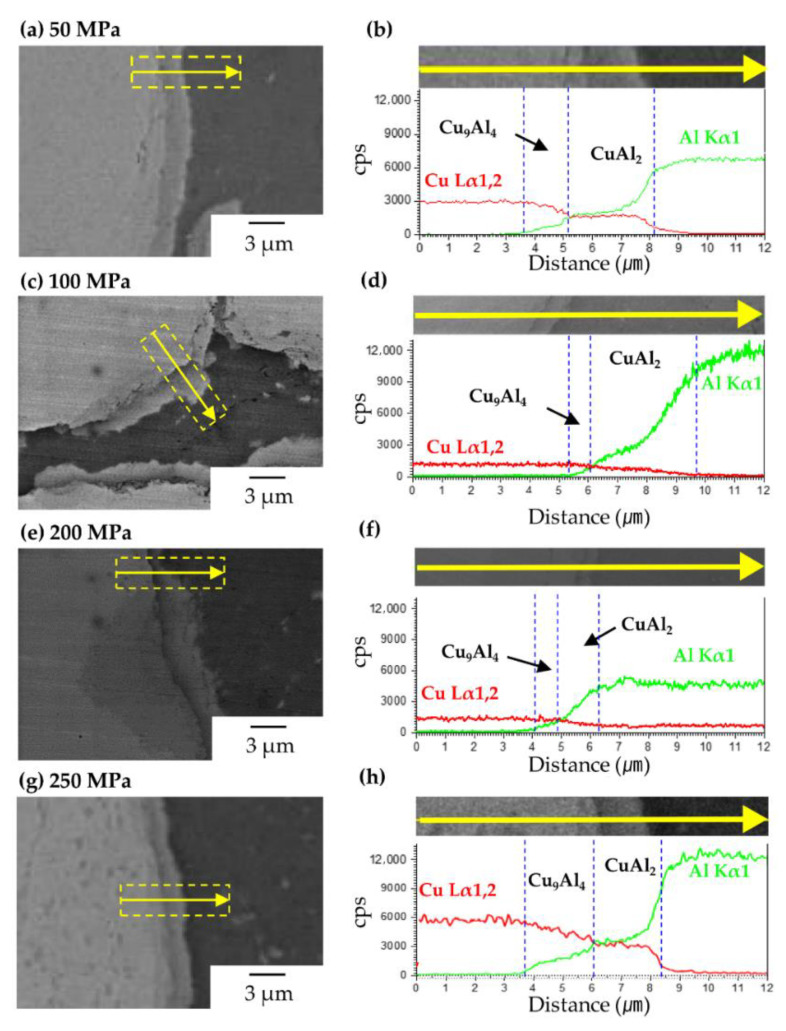
FE-SEM micrograph and EDS line scan result, respectively, for Al/Cu interfaces of composites sintered at (**a**,**b**) 50, (**c**,**d**) 100, (**e**,**f**) 200, and (**g**,**h**) 250 MPa.

From the EDS line scan results shown in Figure 4, the Cu and Al peak intensities decreased towards the Al/Cu interfaces, indicating that Al and Cu had diffused and formed Al–Cu ICs. In the graph of the EDS line scan, the regions of CuAl_2_ and Cu_9_Al_4_ were considered the Al-rich (where in the peak intensity of Al was stronger than that of Cu) and Cu-rich regions (wherein the peak intensity of Cu was stronger than that of Al), respectively. The IC thickness ratio of CuAl_2_ to Cu_9_Al_4_ (see Table 1) changed with pressure. In the Al–Cu composites sintered at 50 MPa (Figure 4b) and 100 MPa (Figure 4d), the diffusion distance of Cu to Al was much greater than of Al to Cu, i.e., the thickness of the CuAl_2_ layer was much greater than that of the Cu_9_Al_4_ layer. However, the thickness ratio of CuAl_2_ to Cu_9_Al_4_ decreased with the increase in pressure, and became nearly 1 at compaction pressures of 200 MPa (Figure 4f) and 250 MPa (Figure 4h). When ICs are formed via thermal diffusion, the CuAl_2_ layer is thicker than the Cu_9_Al_4_ layer. Therefore, there was another dominant mechanism for the formation of ICs under pressure.

Yu et al. reported the formation of interfacial Al–Cu ICs via cold rolling [18]. According to their report, the dislocations accumulated near the interfaces owing to compaction pressure weakened the inter-atomic bonding forces between the individual Al (or Cu), leading to their higher diffusivity [27,28]. Thus, it was concluded that in the present work, the ICs in the Al–Cu composites sintered at approximately 100 MPa were formed via thermal diffusion, but those in the Al–Cu composites sintered at 200–250 MPa were formed via deformation. In addition, the ICs resulting from the application of pressure were only partially formed owing to the uniform accumulation of dislocations along the interface. To compare the dispersion of the ICs formed in the Al–Cu composites sintered at different values of pressure, we observed the cross-sections of the specimens using light microscopy and introduced a colour threshold using the ImageJ program.

**Figure 5:**
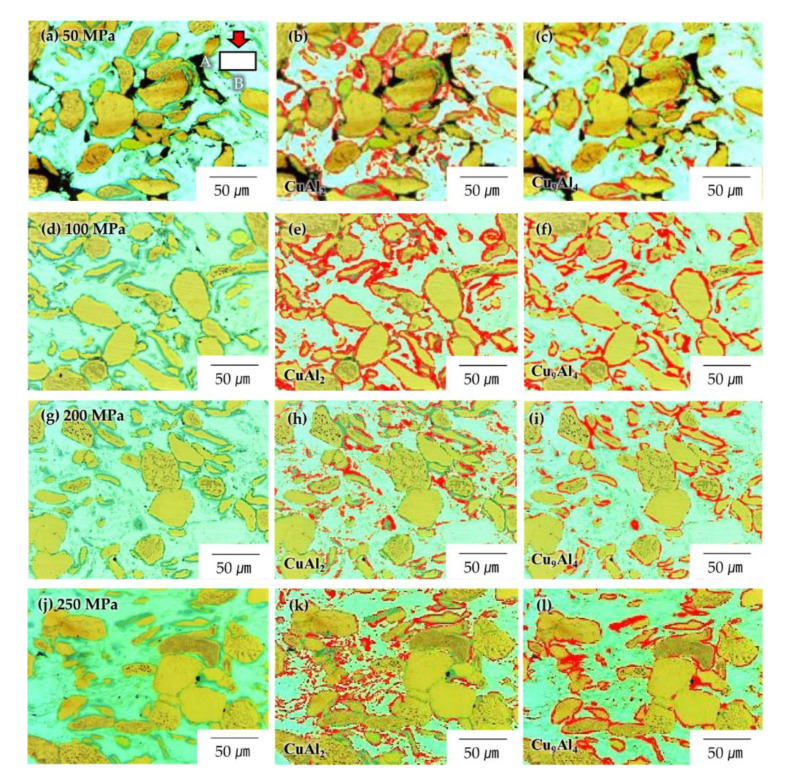
Area fractions of CuAl_2_ and Cu_9_Al_4_, respectively, in Al–50vol%Cu composites sintered at 380 °C and pressure values of (**a**–**c**) 50, (**d**–**f**) 100, (**g**–**i**) 200, and (**j**–**l**) 250 MPa.

Figure 5 shows the light micrographs of the composites, wherein the regions marked in red are the IC formation sites in the Al–50vol%Cu composites fabricated via SPS at 50 MPa (Figure 5a–c), 100 MPa (Figure 5d–f), 200 MPa (Figure 5g–i), and 250 MPa (Figure 5j–l). The area fractions of Al, Cu, CuAl_2_, and Cu_9_Al_4_ were measured (see Table 1). In the Al–Cu composite sintered at 50 MPa, the ICs were only partially formed because the Al/Cu interface was not discontinuous owing to the pores residing between Al and Cu. In the Al–Cu composite sintered at 100 MPa, the pores were nearly removed, and the ICs formed had surrounded the Cu particles uniformly. However, the partial formation of ICs was once again observed in the composite sintered at 200 MPa, although the Al/Cu interface was wholly formed without any pores, as shown in Figure 5e,f. In the Al–Cu composite sintered at the maximum pressure of 250 MPa, the partial formation of ICs became more pronounced and was predominant at the Al/Cu interface vertical to the compression direction (Figure 5g–i). As mentioned previously, the atomic diffusion for the formation of ICs can be driven by either thermal energy or weak atomic bonding owing to severe deformation. The activation energy for atomic diffusion in the Arrhenius equation is decreased by the lattice distortion energy. In this study, heat was applied to the Al–Cu composites, causing severe deformation. The thermal energy was likely used for the recovery of dislocations [29].

As the sintering was conducted under uniaxial pressure, the degree of deformation varied with direction. The Al/Cu interface perpendicular (B) to the application direction of the compressive pressure might have been more severely deformed than that parallel (A) to the direction of applied pressure, as shown in Figure 5a. Therefore, the Al/Cu interfaces might have had different dislocation densities depending on their orientations relative to the direction of compression. When the Al–Cu composites were sintered, the thermal energy was consumed for the recovery of the dislocations near the Al/Cu interfaces. The thermal energy was sufficient to recover only dislocations near interfaces with rather low dislocation densities, whereas dislocations remained at interfaces with high dislocation densities during sintering, and only the latter were involved in the formation of ICs.

**Figure 6:**
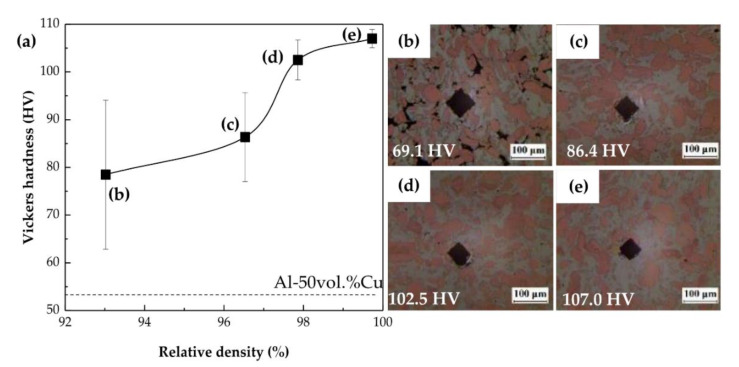
(**a**) Vickers hardness of Al–50vol%Cu composites sintered at 380 °C as a function of relative density, and hardness indentation micrographs of composites sintered at pressures of (**b**) 50, (**c**) 100, (**d**) 200, and (**e**) 250 MPa.

The Al–Cu ICs, with considerably higher hardness than those of Al and Cu, were expected to influence the hardness of the Al–Cu composites significantly. Figure 6 shows the Vickers hardness values of the Al–Cu composites as a function of their relative density. The measured hardness of the Al–Cu composites increased from 78 HV to 108 HV with an increase in relative density, and these values were much higher than the expected value of 53 HV obtained by applying the rule of mixtures to Al and Cu regardless of ICs. In particular, the hardness increased suddenly as relative density increased from 97% to 98%. These results were attributed to the formation of ICs. As compaction pressure changed from 100 MPa to 200 MPa, the main driving force for the formation of ICs changed from thermal diffusion to diffusion by distortion. Hence, the ratio of the amount of Cu_9_Al_4_ to that of CuAl_2_ formed in the Al–Cu composite changed altogether. The Vickers hardness of Cu_9_Al_4_ (549 HV) was nearly 1.5 times that of CuAl_2_ (324 HV) [21,25].

The Al–Cu composite sintered at 250 MPa achieved full densification, i.e., a relative density of 100%, and had a Vickers hardness of 108 HV (see Table 2), which agreed with the value calculated by applying the rule of mixtures to Al, Cu, CuAl_2_, and Cu_9_Al_4_ (Table 1). The amounts of the ICs formed primarily determined the mechanical strength of the Al–Cu composites and were estimated by analysing their area fractions using the colour thresholds.

In addition, as hardness is related to plastic deformation, it is significantly sensitive to the amount of dislocations accumulated inside the composite. This indicates that the accumulation of dislocations was not sufficient to influence the strength of the Al–Cu composites. This supported the theory that the accumulated dislocations were consumed by the application of thermal energy or in the formation of ICs, as mentioned previously. The dislocations were mainly related to the formation behaviour of the ICs. Therefore, further studies on dislocations, including observations at the atomic scale via transmission electron microscopy (TEM), will be conducted.

**Figure 7:**
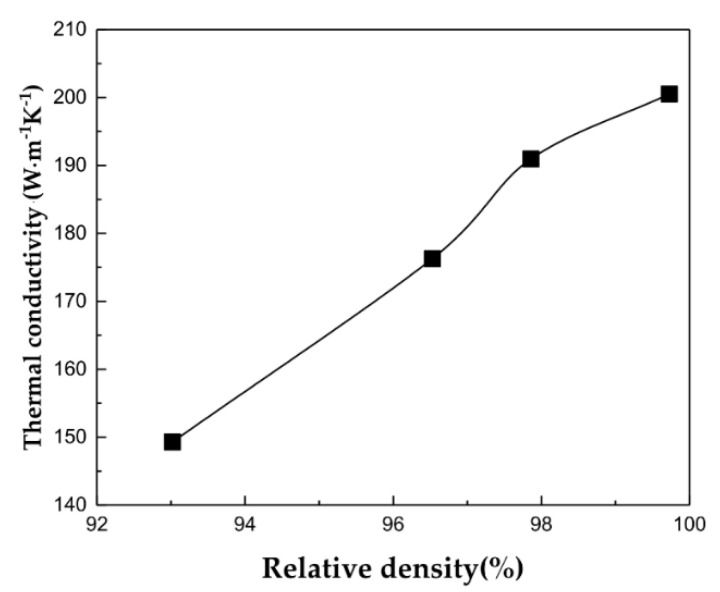
Thermal conductivity of Al–50vol%Cu sintered at 380 °C under various pressure as a function of relative density.

Finally, Al–Cu composites with direct bonded interfaces, controlled formation of ICs, and without accumulation of dislocations were prepared in this study. These IC-controlled Al–Cu composites exhibited improvements in thermal conductivity, which was otherwise deteriorated by the ICs formed, as well as in defects such as dislocations. Figure 7 shows the thermal conductivity of the Al–50vol%Cu composites sintered at 380 °C under various compaction pressures as a function of relative density. The thermal conductivity increased with relative density and reached 201 W·m^−1^·K^−1^ at a relative density of 100% (Table 2). This value was approximately 1.5 times that (130 W·m^−1^·K^−1^) of the Al–50vol%Cu composite sintered at 520 °C under 50 MPa in a previous study [17].

The thermal conductivity of metals is mainly determined by the mobility of free electrons, i.e., the electrical conductivity and thermal conductivity of metals are significantly correlated. Therefore, the thermal conductivity of the Al–Cu composites were calculated using the Wiedemann–Franz law (Equation (1)):

(1) $$K_{e}=\frac{\pi^{2}}{3}{\left(\frac{K_{B}}{e}\right)}^{2}\sigma T,$$

where $K_{e}$ is thermal conductivity, $K_{B}$ is the Boltzmann constant, e is electron charge, σ is electrical conductivity, and T is the absolute temperature. When ICs are formed at the Al/Cu interface, the free electrons cannot pass through the IC layer and the electron mean free path becomes shorter owing to scattering near the interface. The electrical conductivity exponentially decreases with an increase in the thickness of ICs at the interface [30]. As the area fraction of the interface is the highest when the volume fractions of Al and Cu are equal (Al–50vol%Cu), the thermal conductivity decreases [11]. However, in the Al–50vol%Cu composites prepared in the present study, the ICs were controlled to be formed partially at the Al/Cu interfaces via unstable deformation. Hence, the direct bonded Al/Cu interfaces remained fully densified. At the direct contact regions of the Al/Cu interfaces, the electrons might not have been scattered, thereby preventing deterioration in thermal conductivity.

The Al–Cu composites could be densified, forming a partial Al/Cu interface without ICs, which improved their thermal conductivity. Thus, Al–Cu composites of high strength and high thermal conductivity were successfully fabricated using high-pressure SPS. The Al–Cu matrix in such composites can be reinforced with nano-sized fibres such as SiC, CNTs, graphene, and diamond to obtain excellent thermal properties, rendering the materials suitable for use in thermal management components such as heat sinks and printed circuit boards, and in electric device components such as wires and semiconductors.

## 4. Conclusions

In this study, Al–50vol%Cu composites with controlled IC formation were successfully prepared via ball milling followed by high-pressure SPS. First, we optimised the ball-milling process to prepare an Al–Cu composite powder with Cu particles surrounded by Al to achieve uniform dispersion of the former in the latter. This contributed to an increase in the area fraction of the Al/Cu interface. Al–Cu ICs such as CuAl_2_ and Cu_9_Al_4_ were formed during sintering. The expected formation degree of the ICs decreased with an increase in compaction. We systemically investigated the formation mechanism of ICs at the interface under the application of heat energy and physical pressure. When the ICs were formed mainly by the application of thermal energy, they resided throughout the Al/Cu interfaces and their growth entailed considerable consumption of Al, which significantly deteriorated the thermal conductivity of the Al–Cu composites. On the other hand, when ICs generated primarily by distortion resulting from the application of high pressure, they were only partially formed along the interfaces. The regions and amounts of the ICs formed could be controlled by controlling the process conditions such as pressure and temperature. The deterioration in thermal conductivity owing to electron scattering by the ICs at the interfaces was suppressed by the formation of direct bonded interfaces, and the Al–50vol%Cu composite achieved a thermal conductivity of up to 201 W·m^−1^·K^−1^, which was almost equal to that of pure Al. In addition, the formation of ICs might be controlled by varying the temperature, pressure, and even application direction of pressure. This study establishes the possibility of employing powder metallurgy to yield Al–Cu composites with high thermal conductivity.

In addition, it was expected that partially formed ICs could generate strong bonds between Al and Cu and enhance the mechanical strength of the composite based on the two-fold improvement observed in the Vickers hardness test results. In future work, we will evaluate the mechanical properties of the composites as well as undertake atomic-scale observations via TEM to investigate dislocation dispersion, which was the expected driving force in the formation of ICs under high pressure, and investigate the effects of this mechanism on the mechanical and thermal properties of the resultant composites. In conclusion, the Al–Cu matrix composites prepared via powder metallurgy could realise undeteriorated thermal conductivity with high strength in this study, and can be considered for use in thermal management parts such as heat sinks and printed circuit boards, as well as in electric device components such as wires and semiconductors. The controllability of ICs can further enhance the mechanical and thermal properties of the Al–Cu composites.

## Figures and Tables

**Table 1:** Area fractions of Al, Cu, CuAl_2_, and Cu_9_Al_4_, and the thicknesses of CuAl_2_ and Cu_9_Al_4_ layers at the Al/Cu interface in Al–50vol%Cu composites sintered at 380 °C under various pressures.

Compaction Pressure (MPa)	Area Fraction of Phase (%) ± 0.2				Thickness of IC Layers (μm) ± 0.3	
	Al	Cu	CuAl_2_	Cu_9_Al_4_	CuAl_2_	Cu_9_Al_4_
50	40.0	45.8	9.2	5.0	2.9	1.3
100	42.2	41.4	10.0	6.4	3.5	1.0
200	46.4	39.4	5.6	8.6	2.2	2.3
250	47.8	39.0	4.0	9.2	1.8	2.9

**Table 2:** Mechanical and thermal properties of Al–50vol%Cu sintered at 380 °C under various pressures.

Compaction Pressure (MPa)	Relative Density (%)	Vickers Hardness (HV)	Thermal	
			Diffusivity (mm^2^·s^−1^)	Conductivity (W·m^−1^·K^−1^)
50	93.0 ± 2.0	78.5 ± 15.6	42.8	149.3
100	96.5 ± 1.8	86.4 ± 9.3	48.7	176.3
200	97.9 ± 1.3	102.5 ± 4.2	52.1	190.9
250	99.7 ± 0.5	107.0 ± 1.9	53.6	200.5

## Data Availability

Data sharing is not applicable to this article.
